# YiQiFuMai Powder Injection Ameliorates Cerebral Ischemia by Inhibiting Endoplasmic Reticulum Stress-Mediated Neuronal Apoptosis

**DOI:** 10.1155/2016/5493279

**Published:** 2016-03-20

**Authors:** Guosheng Cao, Huana Zhou, Nan Jiang, Yuwei Han, Yang Hu, Yuanyuan Zhang, Jin Qi, Junping Kou, Boyang Yu

**Affiliations:** Jiangsu Key Laboratory of TCM Evaluation and Translational Research, Department of Complex Prescription of TCM, China Pharmaceutical University, Nanjing 211198, China

## Abstract

YiQiFuMai (YQFM) powder injection as a modern preparation derived from Sheng Mai San, a traditional Chinese medicine, has been widely used in the treatment of cardiovascular and cerebrovascular diseases. However, its neuroprotective effect and underlying mechanism in cerebral ischemia remain to be explored. The present study was designed to investigate the neuroprotective effect of YQFM on endoplasmic reticulum (ER) stress-mediated neuronal apoptosis in the permanent middle cerebral artery occlusion- (MCAO-) injured mice and the oxygen-glucose deprivation- (OGD-) induced pheochromocytoma (PC12) cells. The results showed that single administration of YQFM (1.342 g/kg, i.p.) could reduce the brain infarction and improve the neurological deficits and the cerebral blood flow (CBF) after MCAO for 24 h in mice. Moreover, incubation with YQFM (100, 200, and 400 *μ*g/mL) could increase the cell viability, decrease the caspase-3 activity, and inhibit the cell apoptosis in OGD-induced PC12 cells for 12 h. In addition, YQFM treatment could significantly modulate cleaved caspase-3 and Bcl-2 expressions and inhibit the expressions of ER stress-related marker proteins and signaling pathways* in vivo* and* in vitro*. In conclusion, our findings provide the first evidence that YQFM ameliorates cerebral ischemic injury linked with modulating ER stress-related signaling pathways, which provided some new insights for its prevention and treatment of cerebral ischemia diseases.

## 1. Introduction

Ischemic stroke is a devastating disease that ranks secondly after cardiac ischemia as a cause of death and permanent disability worldwide, estimated by the World Health Organization [1]. Since a series of complexed mechanisms are involved in the pathology [2], investigations on the specific and potent treatment for the neuronal functional recovery after cerebral ischemic stroke were extremely limited [3]. According to recent studies, endoplasmic reticulum (ER) stress is one of the essential signaling mechanisms during neuronal injury resulting from cerebral ischemia [4–7]. In response to ischemia or oxygen-glucose deprivation (OGD), ER appears swollen, leading to calcium ion disorder and cell apoptosis through caspase-12 activation [8, 9].

Glucose-regulated protein 78 (GRP78) is a main ER molecular chaperone, regulating protein folding and facilitating protein translocation and protein secretion in ER. It is widely used as a sentinel marker for ER stress under pathologic conditions [10]. C/EBP homologous protein (CHOP) is the first protein identified that promotes apoptosis when ER stress happens. And it is reported to induce apoptosis through downregulating the antiapoptotic factor, Bcl-2 [11]. Activating transcription factor-4 (ATF4) is a stress responsive gene, which is also known as an important mediator of the unfolded protein response (UPR). When ATF4 is ubiquitously expressed at low basal level, its initiation of translation at the authentic start codon is dependent on the upregulated phosphorylation of eIF2*α* by ER stress [12]. Moreover, the expressions of ER stress-target genes, such as CHOP and X-box-binding protein-1 (XBP-1), are also modulated by an ER-membrane-bound transcription factor, activating transcription factor-6 (ATF-6) [13, 14]. Abundant evidence has shown that the inhibition of all three ER stress-related pathways and the cell apoptosis exerts a remarkable protective effect on cerebral ischemia and therefore might be a novel and effective treatment after ischemic stroke [8, 15, 16].

YiQiFuMai (YQFM) powder injection is a modern preparation based on a well-known complex prescription, Sheng Mai San, which is composed of* Panax ginseng* C. A. Mey,* Ophiopogon japonicus* (Thunb.) Ker-Gawl., and* Schisandra chinensis* (Turcz.) Baill. (1 : 3 : 1.5). It was approved in 2007 for treatment of microcirculatory disturbance-related diseases by the China Food and Drug Administration in China [17]. Pharmacological studies have demonstrated that YQFM exerts its beneficial effects on myocardial, vascular, or intestine injuries mainly through inhibiting oxidative damage, NF-*κ*B activation, and cytokines expressions [17–19]. In addition, our network pharmacological studies have also indicated that multiple antiapoptotic pathways and ER stress-related signaling pathways are possibly involved in the mechanism of YQFM against cardiocerebral ischemia diseases [20]. Furthermore, YQFM has been confirmed to ameliorate blood-brain barrier (BBB) dysfunction and brain edema induced by focal cerebral ischemia-reperfusion in mice [21]. However, the effect and potential mechanisms of YQFM on the neuronal apoptosis or oxidative injury remain to be explored.

Given the key role of ER stress-mediated neuronal apoptosis in ischemic stroke [6], the present study is designed to further verify the protective effect of YQFM in permanent middle cerebral artery occlusion- (MCAO-) injured mice and OGD-induced differentiated rat pheochromocytoma (PC12) cells and to explore the involvement of ER stress-related signaling pathways in its effect on neuronal apoptosis. Our results would confirm the efficacy of YQFM in ischemic stroke and may provide some novel insights for its prevention and treatment of cerebral ischemia diseases.

## 2. Materials and Methods

### 2.1. Reagents

YQFM was purchased from Tasly Pharmaceutical Co., Ltd. (Tianjin, China, batch number 20121210). Dantrolene (Dan) was purchased from Sigma (St. Louis, MO, USA). Hoechst 33342 (bisbenzimide) and enhanced chemiluminescence (ECL) reagents were obtained from Beyotime Biotechnology (Shanghai, China). Polyvinylidene fluoride (PVDF) membranes were purchased from Millipore (Bedford, MA, USA). 3-(4,5-Dimethylthiazol-2-yl)-2,5-diphenyltetrazolium bromide (MTT) was purchased from Ameresco (Ameresco, OH, USA). The primary antibodies, horseradish peroxidase- (HRP-) conjugated goat anti-rabbit and anti-mouse IgG, were purchased from Bioworld Technology, Inc. (St. Louis Park, MN, USA).

### 2.2. Animals

Male C57BL/6J mice weighing 18–22 g were obtained from the Model Animal Research Centre of Yangzhou University (Yangzhou, China, certificate number SCXK 2014-0004). All procedures and assessments were approved by the Animal Ethics Committee of the School of Chinese Materia Medica, China Pharmaceutical University. The experiments were carried out in accordance with the National Institutes of Health Guide for the care and use of laboratory animals (NIH Publication number 80-23, revised 1996). Mice were housed in a 12 h light-dark cycle and allowed free access to food and water. Prior to experiments, all animals were randomized into experimental groups and measured blindly.

### 2.3. Focal Cerebral Ischemia

C57BL/6J mice were subjected to MCAO as reported previously [22]. Briefly, animals were anesthetized with 4% chloral hydrate (0.1 mL/10 g body weight) intraperitoneally (i.p.), after a midline incision of the neck skin, and branches of the right external carotid artery were isolated and cauterized. In brief, a 6-0 nylon monofilament suture, blunted at the tip and coated with 1% poly-L-lysine, was advanced 9-10 mm into the internal carotid to occlude the origin of the middle cerebral artery (MCA) for 24 h. Body temperature was maintained with a heating pad (Alcbio; Shanghai, China) at 37.0 ± 0.5°C during operation and ischemia. Sham-operated animals underwent the same surgical procedure, but the suture was not advanced into the internal carotid artery. A laser Doppler flow meter (LDF; FLPI2, Moor, England) was used to confirm the decrease of the blood flow immediately after the occlusion to below 30% compared with nonischemia side.

### 2.4. Cell Culture

Nerve growth factor- (NGF-) differentiated rat adrenal pheochromocytoma (PC12) cells were obtained from the Shanghai Institute of Cell Biology, Chinese Academy of Sciences. Cells were cultured in Dulbecco's modified Eagle's medium (DMEM, Gibco, NY, USA) with 10% fetal bovine serum (FBS, PAA, Queensland, Australia), 100 U/mL penicillin, and 100 U/mL streptomycin (Ameresco, OH, USA), at 37°C in a humidified atmosphere of 5% CO_2_ and 95% air. The growth medium was changed every other day, and cells were inoculated on 96-well plates or Petri dishes at a density appropriate to the experimental requirements.

### 2.5. Oxygen-Glucose Deprivation (OGD) and Drug Treatment

The PC12 cell cultures were subjected to OGD for 12 h by incubation in a deoxygenated glucose-free balanced salt solution. The cells were cultured in the DMEM medium without glucose and incubated at 37°C in a humidified atmosphere of 5% CO_2_, 94% N_2_, and 1% O_2_. There were six different groups in the experiments: control, OGD exposure (model), various concentrations of YQFM (100–400 *μ*g/mL), and the antioxidant Dan (30 *μ*M) as a positive control for 12 h during OGD treatment. YQFM was dissolved in DMEM culture medium without glucose and Dan was dissolved in DMSO (SunShineBio, Nanjing, China) at the final concentration of 0.1%.

### 2.6. Evaluation of Infarct Volume, Neurological Deficits, and Cerebral Blood Flow

To confirm whether YQFM exerts neuroprotective effect* in vivo*, the mice were randomly divided into 4 groups: Sham, Sham + YQFM, MCAO, and MCAO + YQFM. Mice were given YQFM at 1.342 g/kg (equal to 2 times the clinical dose, according to [21]) or equal volume of 0.9% sodium chloride by intraperitoneal administration after MCAO onset. Briefly, brains were quickly removed 24 h after MCAO. TTC staining was performed to measure the infarct volume [23]. Neurological deficit of the experimental animals was assessed according to Longa's method as reported previously [24]. Cerebral blood flow (CBF) was measured by a laser Doppler flowmetry as described previously [25]; the images were acquired at 24 h after ischemia.

### 2.7. Cell Viability

Cell viability was measured by MTT assay as reported previously [26]. Briefly, PC12 cells were plated at a density of approximately 1 × 10^4^/well in 96-well plates and grown in DMEM overnight. Various concentrations of YQFM (100, 200, and 400 *μ*g/mL) or Dan (30 *μ*M) were added in PC12 cells and then exposed to OGD for 12 h. The cells were sequently treated with MTT solution at the final concentration of 0.5 mg/mL in normal environment and fresh medium without YQFM for 4 h at 37°C, followed by the addition of 150 *μ*L of DMSO, and OD values of the 96-well plates were detected with a microplate reader (Epoch, BioTek, USA) after being shaken for 10 min, with a detection wavelength of 570 nm, and a reference wavelength of 650 nm. Cell viability was expressed as a percentage of control group.

### 2.8. Detection of Apoptotic Cells

Hoechst 33342 dye staining was used to detect the nuclear fragmentation for evaluation of cell apoptosis according to the methods described [27]. PC12 cells were seeded and treated in 24-well plate. After the treatment, the cells were stained with Hoechst 33342 (1 *μ*g/mL) for 30 min at 4°C and washed with PBS twice. Then, the cells were visualized using a fluorescence microscope. Nuclear morphological changes characteristic of apoptosis such as fragmented or condensed nuclei with strong bright blue color were observed.

Cell apoptosis was also tested with Annexin V-FITC and PI staining followed by analysis with flow cytometry, according to the instruction of the Annexin V-FITC/PI detection kit (BD Biosciences, USA). Briefly, the treated cells were washed with cold PBS and dissociated using trypsin and centrifuged at 2,000 rpm for 5 min. Then, the cells were stained with 5 *μ*L of Annexin V-FITC and 5 *μ*L of PI resuspended in Annexin V binding buffer (100 *μ*L) at room temperature in the dark for 10 min. The cells were then analyzed by flow cytometry using FL1 channel for fluorescein detection and FL3 channel for PI detection.

### 2.9. Measurement of Caspase-3 Activity

PC12 cells were pretreated with YQFM (100, 200, and 400 *μ*g/mL) and Dan (30 *μ*M) when exposed to OGD for 12 h. The caspase-3 activity was detected according to the manufacturer's instructions of assay kit (Beyotime, Shanghai, China) as previously [28]. The absorbance was detected by a microplate reader at 405 nm. Protein levels in the supernatant were measured by Bradford method. The results were expressed as active units of caspase-3 per mg protein.

### 2.10. Western Blot Analysis

Western blotting analyses were performed as described previously [29]. The cells and brain tissue were lysed in the buffer supplemented with protease inhibitor and centrifuged at 12,000 rpm for 10 min at 4°C after washing with ice-cold PBS. Equal amounts of proteins (30 *μ*g) were loaded into a 12.5% SDS-PAGE and transferred to PVDF membranes (Millipore Corporation, USA) by electrophoresis. After blocking with 5% BSA for 1.5 h, PVDF membranes were probed overnight at 4°C with primary antibodies against cleaved caspase-3 (1 : 800, CST, USA), Bcl-2 (1 : 1000, CST, USA), GRP78 (1 : 5000, Sigma, USA), CHOP (1 : 1000, CST, USA), caspase-12 (1 : 1000, Bioworld, USA), ATF-6 (1 : 500, Bioworld, USA), ATF-4 (1 : 500, Bioworld, USA), XBP-1 (1 : 1000, Bioworld, USA), eIF2*α* (1 : 500, Bioworld, USA), phospho-eIF2*α* (1 : 500, Bioworld, USA), and *β*-actin (1 : 2000, Bioworld, USA). The blots were then incubated with horseradish peroxidase- (HRP-) conjugated anti-rabbit or anti-mouse secondary antibody (dilution 1 : 8,000) and developed with ECL reagent. The immune-reactive bands were visualized using the ChemiDoc*™* MP System (Bio-Rad, California, USA) and the results were quantified by using the Image Lab*™*Software (version 4.1, Bio-Rad).

### 2.11. Statistical Analysis

All results are expressed as the mean ± standard deviation. Statistical analysis was carried out using Student's two-tailed *t*-test for comparison between two groups and one-way analysis of variance (ANOVA) followed by Dunnett's test when the data involved three or more groups. *P* < 0.05 was considered statistically significant. All analyses were performed with GraphPad Prism Version 5.01 (GraphPad Software Inc., USA).

## 3. Results

### 3.1. Effects of YQFM on the Infarct Size, Neurologic Deficit Scores, and Cerebral Blood Flow (CBF) in MCAO-Injured Mice

**Figure 1:**
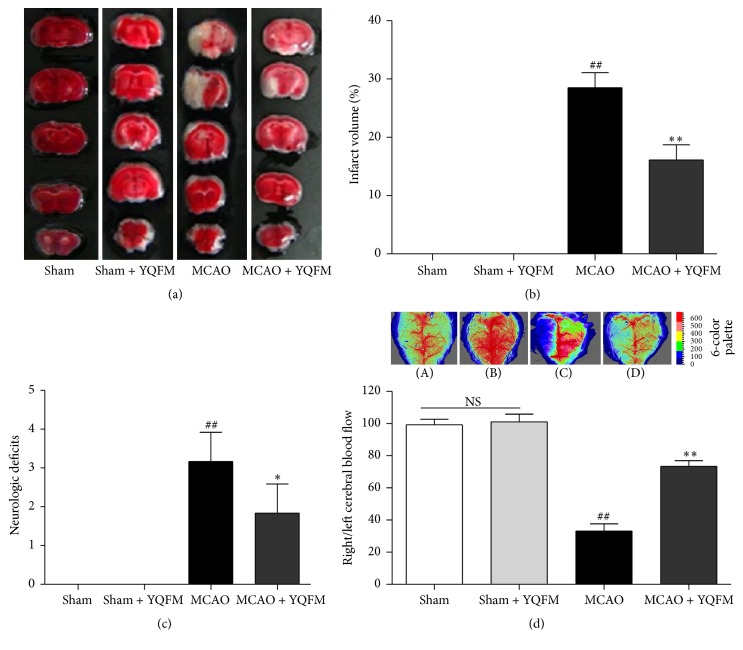
Effects of YQFM on infarct size, neurologic deficit scores and cerebral blood flow in MCAO-injured mice. Mice were subjected to 24 h cerebral ischemia. YQFM (1.342 g/kg) was given by intraperitoneal administration after ischemia. (a) Representative TTC-stained brain sections in different groups. (b) Quantitative analysis of infarct volume in different groups. (c) Quantitative analysis of neurologic deficit scores in different groups. (d) The magnitude of CBF is represented by different colors and quantitative analysis of CBF in different groups. Data were presented as mean ± SD. ^##^ *P* < 0.01 versus Sham, ^*∗*^ *P* < 0.05, ^*∗∗*^ *P* < 0.01 versus MCAO (*n* = 6~8).

Infarct volume, neurological deficit, and cerebral blood flow were evaluated 24 h after MCAO. TTC-staining image and quantitative analysis of infarct volume demonstrated that the group of YQFM at the dosage of 1.342 g/kg had much smaller infarct size than that in the MCAO group (Figures 1(a) and 1(b)). After MCAO, the neurological deficit score of the mice in MCAO + YQFM group was significantly decreased comparing to the MCAO group, which indicated that YQFM could improve neurological deficit after 24 h MCAO in mice as described in Figure 1(c). In addition, CBF was measured by a laser Doppler flowmetry; the result demonstrated that administration of YQFM at the dosage of 1.342 g/kg resulted in a significant increase on CBF 24 h after MCAO (Figure 1(d)), which indicated that the smaller infarct volume in YQFM-treated groups was correlated with improved CBF during MCAO. No obvious change was observed between Sham and Sham + YQFM groups in above experiments (Figure 1).

### 3.2. Effect of YQFM on ER Stress-Related Proteins in MCAO-Injured Mice

**Figure 2:**
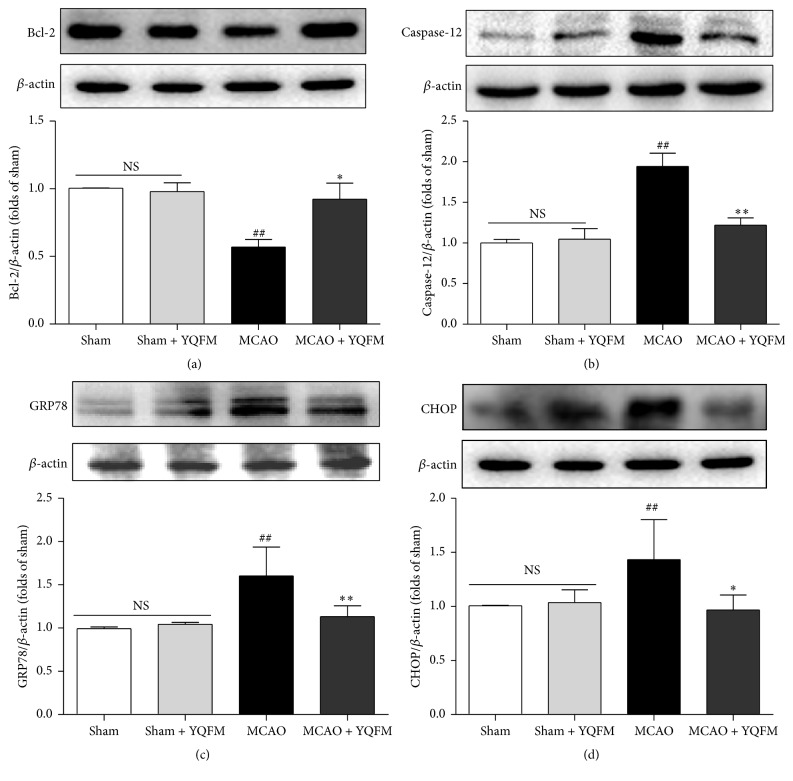
Effects of YQFM on ER stress-related proteins in MCAO-injured mice. Mice were subjected to 24 h of cerebral ischemia, and YQFM (1.342 g/kg) was given by intraperitoneal administration after ischemia. Representative Western blot and the quantitative analysis of the ratio of (a) Bcl-2, (b) caspase-12, (c) GRP78 and (d) CHOP. Data were presented as mean ± SD. ^##^ *P* < 0.01 versus Sham, ^*∗*^ *P* < 0.05, and ^*∗∗*^ *P* < 0.01 versus MCAO (*n* = 3).

The expressions of ER stress-related marker proteins and signaling pathways were examined with Western blot. As demonstrated in Figure 2, the administration of YQFM (1.342 g/kg) notably increased the expression of Bcl-2 and decreased the expressions of caspase-12, GRP78, and CHOP. All these data indicate that YQFM improves cerebral ischemic injury linked with ER stress in MCAO mice. No obvious change was observed between Sham and Sham + YQFM groups in above experiments (Figure 2).

### 3.3. Effect of YQFM on ER Stress-Related Signaling Pathways in MCAO-Injured Mice

**Figure 3:**
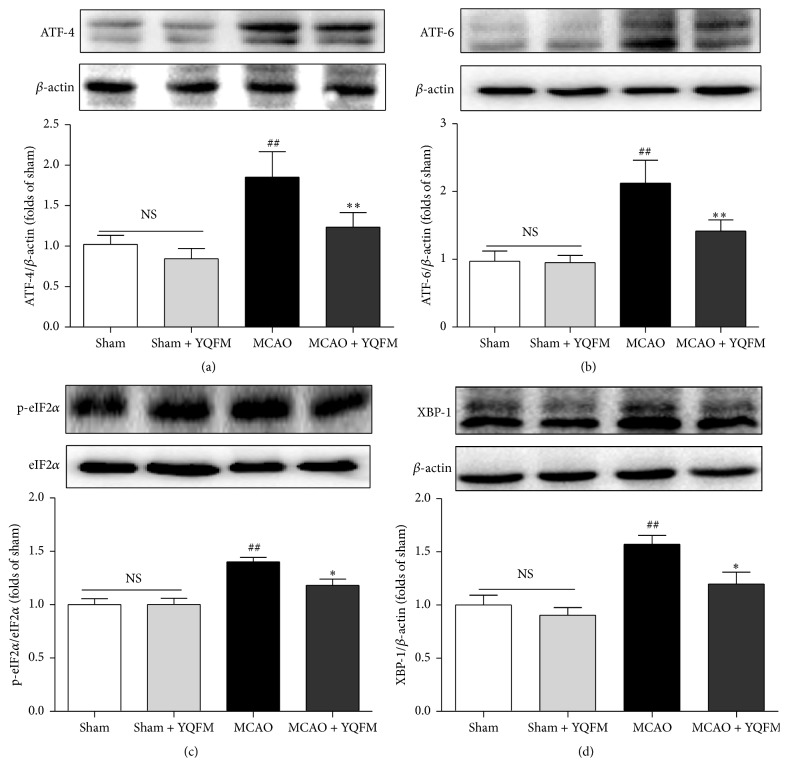
Effects of YQFM on ER stress-related signaling pathways in MCAO-injured mice. Mice were subjected to 24 h of cerebral ischemia, and YQFM (1.342 g/kg) was given by intraperitoneal administration after ischemia. Representative Western blot and the quantitative analysis of the ratio of (a) ATF-4 and (b) ATF-6 and (c) the ratio of p-eIF2*α*/eIF2*α* and (d) XBP-1. Data were presented as mean ± SD. ^##^ *P* < 0.01 versus Sham, ^*∗*^ *P* < 0.05, and ^*∗∗*^ *P* < 0.01 versus MCAO (*n* = 3).

The expressions of ER stress-related signaling pathways were examined with Western blot. The Western blot results demonstrated that YQFM treatment could inhibit the expressions of ATF-4, ATF-6, XBP-1, and phospho-eIF2*α* in MCAO mice. No obvious change was observed between Sham and Sham + YQFM groups in those results. All these data indicate that YQFM improves cerebral ischemic injury linked with ER stress-related signaling pathways in MCAO mice (Figure 3).

### 3.4. Effect of YQFM on OGD-Injured Cell Viability in PC12 Cells

**Figure 4:**
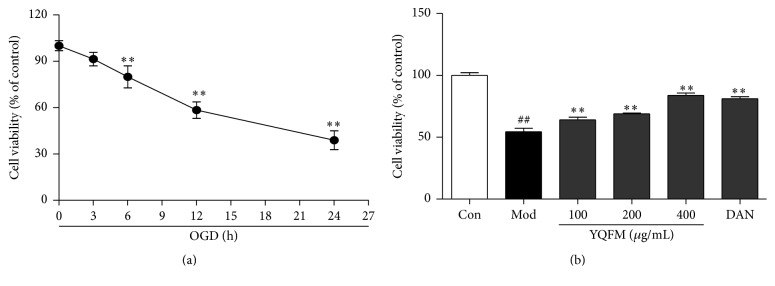
Effect of YQFM on the OGD-injured cell viability in PC12 cells. (a) Cell viability was measured by MTT assay after OGD for 3, 6, 12, 18, 21, 24, and 27 h. (b) PC12 cells were treated with YQFM at the concentrations of 100, 200, and 400 *μ*g/mL for 12 h during OGD. Cell viability was measured by MTT assay. Data were presented as mean ± SD. ^##^ *P* < 0.01 versus control, ^*∗*^ *P* < 0.05, and ^*∗∗*^ *P* < 0.01 versus model (*n* = 6).

We investigated the injurious effects of different time of OGD on PC12 cells using MTT assay. The result demonstrated that the cell viability significantly decreased by approximately 40% when the cultured PC12 cells were exposed to OGD for 12 h (Figure 4(a)). Therefore, we adopted OGD for 12 h in the subsequent experiments. The effect of various concentrations of YQFM (100–400 *μ*g/mL) pretreatment on OGD-injured (12 h explosion) PC12 cells was also investigated with Dan at the dosage of 30 *μ*M as the positive control. The result demonstrated that the OGD-induced reduction of PC12 cell viability was significantly increased by the pretreatment of YQFM in various concentrations of 100–400 *μ*g/mL or Dan (30 *μ*M) (Figure 4(b)).

### 3.5. Effect of YQFM on OGD-Induced Apoptosis in PC12 Cells

**Figure 5:**
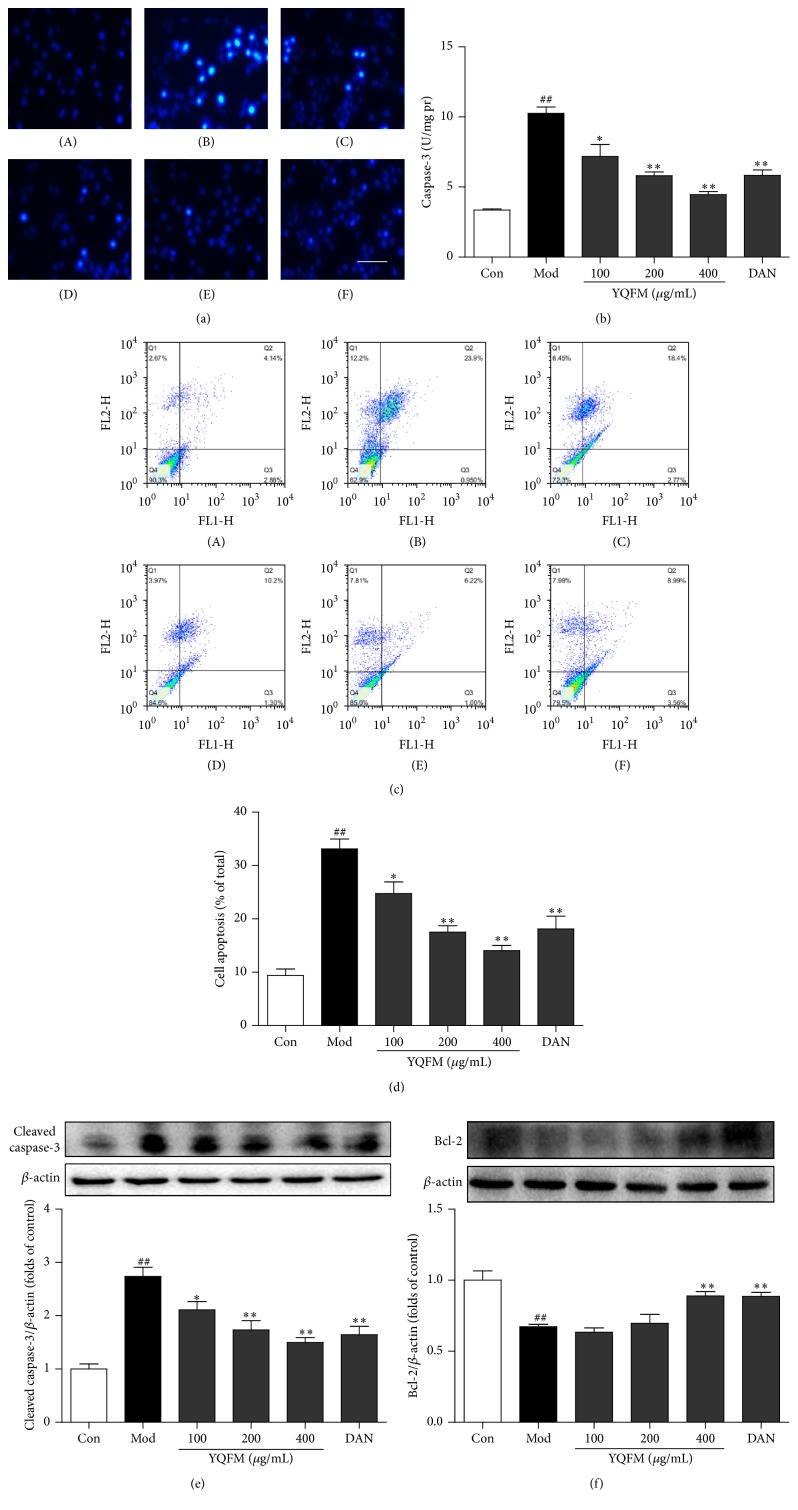
Effect of YQFM on OGD-induced cell apoptosis in PC12 cells. (a) PC12 cells were treated with YQFM (100, 200, and 400 *μ*g/mL) or Dan (30 *μ*M) for 12 h during OGD before Hoechst 33342 staining (200x, final magnification) as follows: control group; model group; group pretreated with YQFM at the concentrations of 100, 200, and 400 *μ*g/mL and then exposed to OGD; group pretreated with 30 *μ*M Dan and then exposed to OGD. Scale bar = 50 *μ*m. (b) Quantitative analysis of caspase-3 activity which was tested by test kit. (c) Cell apoptosis was tested by flow cytometry analysis as follows: control group; model group; group pretreated with YQFM at the concentrations of 100, 200, and 400 *μ*g/mL and then exposed to OGD; group pretreated with 30 *μ*M Dan and then exposed to OGD. (d) The rate of cell apoptosis (%) was analyzed by flow cytometry analysis. The expressions of (e) cleaved caspase-3 and (f) Bcl-2 were detected by Western blot. Blots normalized to *β*-actin expression were shown. Data were presented as mean ± SD. ^##^ *P* < 0.01 versus control, ^*∗*^ *P* < 0.05, and ^*∗∗*^ *P* < 0.01 versus model (*n* = 3).

Whether YQFM could protect against neuronal injury via the inhibition of apoptosis needs to be confirmed. As the Hoechst 33342 staining result demonstrated, the fluorescence intensity was decreased with the presence of the different concentrations of YQFM (100–400 *μ*g/mL) and Dan (30 *μ*M), comparing to the MCAO model group (Figure 5(a)). Correspondingly, when exposed to OGD injury, the activity of caspase-3, final executor of apoptosis, was remarkably elevated, comparing to the control group, while the pretreatment of YQFM (100–400 *μ*g/mL) or Dan (30 *μ*M) significantly decreased the activity of caspase-3 (Figure 5(b)). In addition, Annexin V-FITC/PI was used to detect early apoptotic cells and identify late-stage apoptosis due to its ability to penetrate into the nuclear of apoptotic cells, while it is the opposite in normal cells (from 9.37% to 33.13%). The flow cytometry results demonstrated that the pretreatment of YQFM at the three concentrations or Dan (30 *μ*M) could significantly rescue the PC12 cells exposed for 12 h from apoptosis with the percentage of 24.73%, 17.53%, 14.06%, and 18.13%, respectively. (Figures 5(c) and 5(d)).

Furthermore, we investigated the effects of YQFM on the expression of apoptosis-related proteins, such as cleaved caspase-3 and Bcl-2 in PC12 cells. According to Western blot, the treatment with YQFM or Dan decreased the cleaved caspase-3 expression and increased Bcl-2 expression, comparing to the OGD model group (Figures 5(e) and 5(f)).

### 3.6. Effect of YQFM on the Expression of ER Stress-Related Proteins in OGD-Induced PC12 Cells

**Figure 6:**
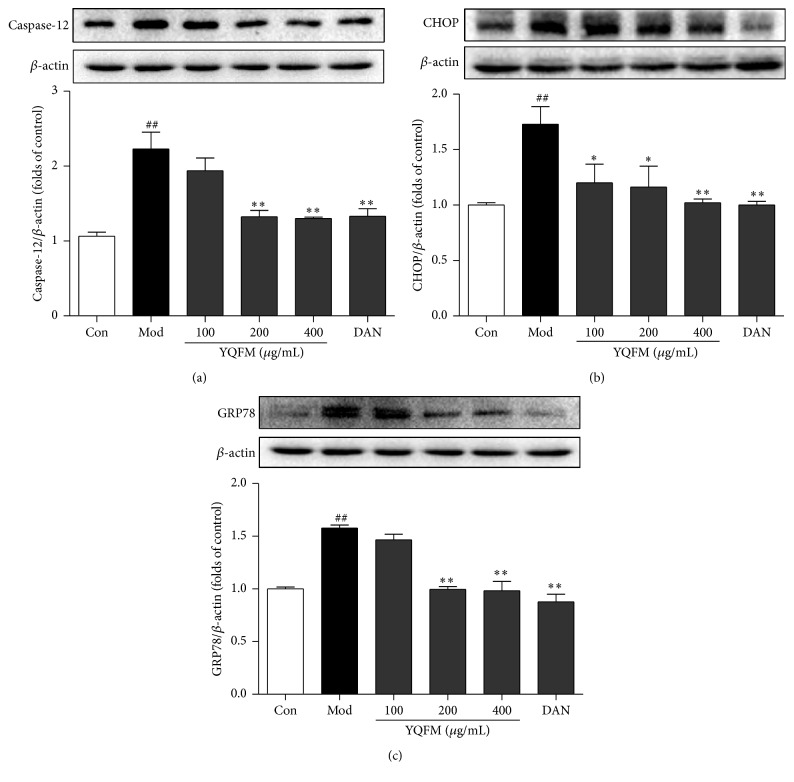
Effects of YQFM on the expression of ER stress-related proteins in OGD-induced PC12 cells. PC12 cells were treated with YQFM (100, 200, and 400 *μ*g/mL) or Dan (30 *μ*M) for 12 h during OGD, the treated cells were lysed, and proteins were isolated and detected by Western blotting. Quantitative analysis of (a) caspase-12, (b) CHOP, and (c) GRP78 was performed by Image Lab*™*Software. Blots normalized to *β*-actin expression were shown. Data were presented as mean ± SD. ^##^ *P* < 0.01 versus control, ^*∗*^ *P* < 0.05, and ^*∗∗*^ *P* < 0.01 versus model (*n* = 3).

To investigate the involvement of ER stress in the neuroprotective effect of YQFM, we measured the ER apoptosis specific activator protein, cleaved caspase-12, and the expressions of ER stress markers, CHOP and GRP78, with Western blot. The results demonstrated that the pretreatment of YQFM at 200 and 400 *μ*g/mL or Dan (30 *μ*M) could inhibit the increased expression of caspase-12, CHOP, and GRP78 in OGD-induced PC12 cells. The percentages of inhibition were 74.35%, 94.66%, and 99.81%, respectively (YQFM (400 *μ*g/mL) versus model) (Figure 6).

### 3.7. Effect of YQFM on ER Stress-Related Signaling Pathways in OGD-Induced PC12 Cells

**Figure 7:**
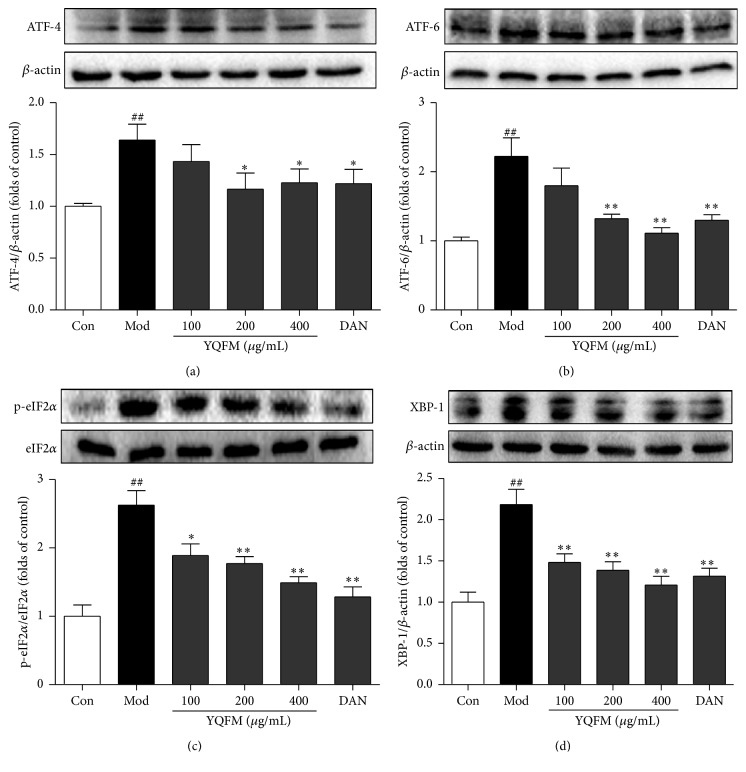
Effects of YQFM on ER stress-related signaling pathways in OGD-induced PC12 cells. PC12 cells were treated with YQFM (100, 200, and 400 *μ*g/mL) or Dan (30 *μ*M) for 12 h during OGD, and then cells were lysed and proteins were isolated. The expressions of (a) ATF-4 and (b) ATF-6 and (c) the ratio of p-eIF2*α*/eIF2*α* and (d) XBP-1 were detected by Western blotting. Blots normalized to *β*-actin expression were shown. Data were presented as mean ± SD. ^##^ *P* < 0.01 versus control, ^*∗*^ *P* < 0.05, and ^*∗∗*^ *P* < 0.01 versus model (*n* = 3).

To explore the underlying mechanism of the inhibition effect of YQFM on ES stress-related neuronal apoptosis, we investigated the effects of YQFM on the UPR-associated genes, such as ATF-4, ATF-6, eIF2*α*, phospho-eIF2*α*, and XBP-1 in PC12 cells. The Western blot results demonstrated that the expressions of ATF-4, ATF-6, XBP-1, and phosphor-eIF2*α* were upregulated when exposed to OGD for 12 h, while the treatment of YQFM at three concentrations significantly downregulated the triggered protein expressions, corresponding to that of the positive drug, Dan (30 *μ*M). The percentage of inhibition was 62.67%, 89.46%, 68.97%, and 81.04%, respectively (YQFM (400 *μ*g/mL) versus model) (Figure 7).

## 4. Discussion

In the present study, we confirmed the neuroprotective effect of YQFM and explored the role of ER stress-mediated neuronal apoptosis in MCAO-injured mice and OGD-injured PC12 cells.* In vivo* results demonstrated that the treatment of YQFM (1.342 g/kg) could reduce brain infarct size, improve neurological deficits and increase CBF, and regulate the expression of ER stress-related proteins and signaling pathways in MCAO mice brain. The* in vitro* results correspondingly indicated that YQFM (100, 200, and 400 *μ*g/mL) could increase the injured cell viability, inhibit cell apoptosis, and regulate the ER stress-related proteins and signaling pathways in OGD-induced PC12 cells.

Series of medicines, such as dantrolene and edaravone, which have been proved to be effective in attenuating ER stress after experimental cerebral ischemia, may become a potent therapeutic agent as the treatment for ischemic brain disease in the near future [5, 15, 30–32]. Dantrolene, used as a ryanodine receptor antagonist, can significantly decrease infarct volume and provide neuroprotective effect on rats after 1.5 h of MCAO and 24 h of reperfusion via reducing ER stress-mediated apoptotic signaling pathways by inhibiting the expressions of eIF2*α* phosphorylation, ATF-4, and CHOP [30]. Thus, dantrolene was used as the positive control in this present study and we confirmed that YQFM indicated similar effect of dantrolene in the ischemic stroke model mice. Moreover, our data further indicated that YQFM could inhibit the expressions of GRP78, caspase-12, ATF-6, and XBP-1 in MCAO-injured mice for 24 h.

ER stress is an essential step in the progression of MCAO injury and plays an important role in ischemic neuronal cell apoptosis as previously reported [16, 33]. Remote ischemic postconditioning can decrease the protein level of phospho-eIF2*α*, caspase-12, and CHOP, which protects against brain injury in rats of MCAO and reperfusion by attenuating ER stress response-induced apoptosis [34]. It is reported that nafamostat mesilate (NM), a type of serine protease inhibitor, can attenuate MCAO and reperfusion-induced brain injury via the inhibition of GRP78, CHOP, and phospho-eIF2*α* [35]. Therefore, modulation on ER stress exerts a remarkable protective effect on the ischemic brain and provides the prospect of new stroke therapies. Here, we verified the protective effect of YQFM and its possible mechanism related with ER stress* in vivo*. According to our data, YQFM could reduce brain infarct size, improve neurological deficits, and increase CBF in MCAO-injured mice, which further confirmed the protective effect of YQFM in ischemic stroke, corresponding to the previous study [18]. Moreover, YQFM also could regulate the expressions of ER stress-related proteins and signaling pathways, modulating the expressions of not only GRP78, caspase-12, CHOP, and phospho-eIF2*α*, but also ATF-4, ATF-6, and XBP-1 (Figures 2 and 3). The results suggested that YQFM could regulate some other ER stress-related pathways in MCAO-injured mice. All these findings demonstrate YQFM can improve MCAO-induced brain damage in mice, and the beneficial effects may be linked with ER stress-related signaling pathways.

In addition, the previous studies have demonstrated that OGD exposure can result in cell viability reduction and cell apoptosis in many different cell types, including neuronal cells, astrocyte, and vascular endothelial cells [36–38]. Furthermore, NGF-differentiated PC12 cells have been extensively used as a neuronal cells line model for the nervous system study, and with regard to the* in vitro* study of hypoxia, an OGD model of PC12 cells has been established [39, 40]. Therefore, we evaluated the efficacy of YQFM in OGD-induced PC12 cells in this present study. We found that incubation with YQFM increased cell viability and decreased the ratio of cell apoptosis by the MTT assay and Hoechst 33342 staining and Annexin V-FITC/PI analysis (Figures 4, 5(a), and 5(b)). Moreover, YQFM could inhibit the expression of cleaved caspase-3 and increase the expression of Bcl-2 (Figures 5(e) and 5(f)), which are the most important apoptosis-related proteins in the process of cell apoptosis [41]. Therefore, our findings indicate YQFM can protect PC12 cells from OGD-induced injury via inhibiting cell apoptosis.

ER stress is a major intracellular signal transduction pathway of apoptosis [11, 33, 42]. Several studies have suggested that ER stress plays a critical role in a variety of processes including ischemia and hypoxia [8, 34, 43]. Moreover, induction of ER stress in response to OGD also involves the activation of the protein kinase RNA- (PKR-) like ER kinase- (PERK-) eIF2*α*-ATF4 and IRE1-XBP1 pathways, and the increased expression of caspase-12, GRP78, and CHOP [8, 11, 44]. Thus, we then pointed to whether attenuation of ER stress-induced apoptotic signaling pathways contributed to the mechanisms in OGD-injured PC12 cells. Our study is in good agreement with these studies that OGD could stimulate ER stress to induce apoptosis in PC12 cells, while YQFM could inhibit the expression of ER stress-related proteins caspase-12, GRP78, and CHOP (Figure 6) and UPR-associated genes ATF-4, ATF-6, phospho-eIF2*α*, and XBP-1 (Figure 7). We also verified that YQFM could modulate the expressions of cleaved caspase-3 and Bcl-2, which was the direct evidence of attenuation of ER stress-mediated apoptotic signaling pathways in OGD-injured PC12 cells. All these findings indicated that YQFM ameliorates OGD-induced neuronal apoptosis linked with modulating ER stress-related signaling pathways.

Apart from these findings, the present study still has several limitations due to many other possible mechanisms existing in the ischemia-induced apoptosis model, such as extrinsic apoptosis pathway and mitochondrial apoptosis pathway [41, 45, 46]. Furthermore, whether or not YQFM can modulate cell apoptosis through other related signaling pathways still remains unclear. In the current study, we evaluated the effects of YQFM on GRP78-XBP1-ATF6-eIF2*α* signaling pathway* in vitro*. However, other signaling pathways related to ER stress need to be further examined. In addition, as previously described [47], YQFM consists of various bioactive compounds, such as ginsenoside Rg1, ginsenoside Rb1, and schisandrin, some of which display neuroprotective effects after OGD injury [26, 48]. Moreover, some reports have demonstrated that those bioactive compounds can inhibit cell apoptosis by inducing ER stress. For example, ginsenoside Rg1 can exhibit neuroprotective effects by inhibiting the ER stress-mediated c-Jun N-terminal kinase (JNK) apoptotic pathway in a rat model of Alzheimer's disease, and ginsenoside Rb1 also can suppress the activation of ER stress-associated proteins including PERK and CHOP, and downregulation of Bcl-2 induced by high glucose [49, 50]. These findings prompted that YQFM could inhibit ER stress-mediated neuronal apoptosis linked with JNK and PERK apoptotic signaling pathways. Therefore, how those bioactive compounds or their combination contributes to the global activities of YQFM needs further investigation. On the other hand, ER stress and oxidative stress are linked to multiple human pathologies, including metabolic, neurodegenerative, immune/inflammatory, and neoplastic diseases [51–53], and whether YQFM could be beneficial for these diseases is of a great interest to explore.

**Figure 8:**
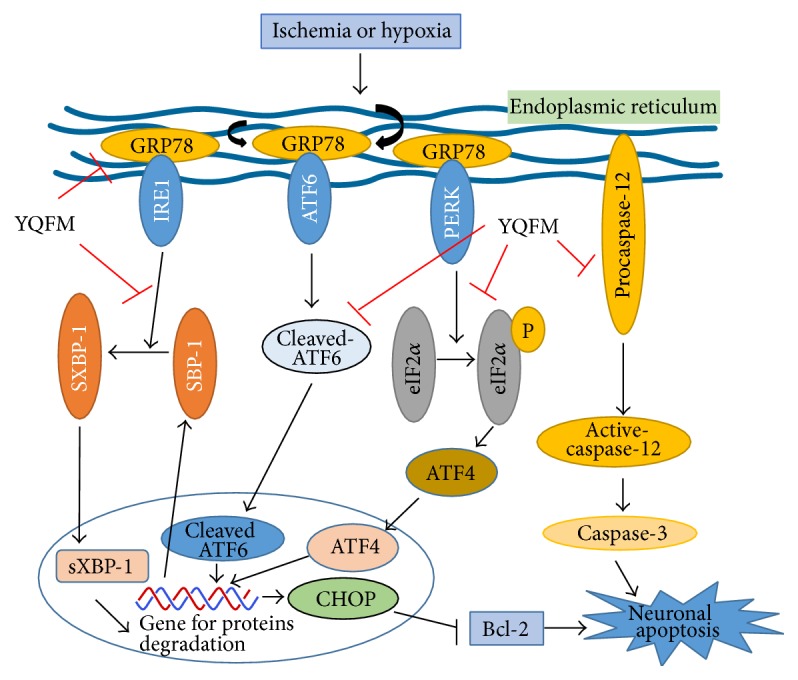
Proposed mechanisms of YQFM on ER stress-mediated neuronal apoptosis in cerebral ischemia injury. Under conditions of physiological homeostasis, PERK, ATF-6, and IRE1 interact with GRP78 in ER. Upon hypoxia or ischemia, upregulated GRP78 activates the autophosphorylation of IRE-1 and PERK and triggers XBP-1, ATF-6, and eIF2*α* phosphorylation. Phosphorylation of eIF2*α* activates the ATF-4. All those three pathways contribute to gene for protein degradation and activate CHOP, which inhibits the antiapoptotic protein, Bcl-2. Moreover, activated caspase-12 activates caspase-3, leading to enhanced neuronal apoptosis and cerebral damage. YQFM inhibits neuronal apoptosis and protects brain damage via modulating ER stress-related signaling pathways.

In conclusion, our findings demonstrate that YQFM treatment provides a protective effect against brain damage induced by ischemia injury* in vivo* and* in vitro*, and the attenuation of ER stress-mediated neuronal apoptosis may be involved in the mechanisms of YQFM (schematic graph is shown in Figure 8). Our findings provide the experimental basis of YQFM for the prevention and treatment of cerebral ischemia disease, enriching the function of YQFM in clinical application.
